# Microbiological Food Safety Surveillance in China

**DOI:** 10.3390/ijerph120910662

**Published:** 2015-08-28

**Authors:** Xiaoyan Pei, Ning Li, Yunchang Guo, Xiumei Liu, Lin Yan, Ying Li, Shuran Yang, Jing Hu, Jianghui Zhu, Dajin Yang

**Affiliations:** National Center for Food Safety Risk Assessment, Beijing 100021, China; E-Mails: peixiaoyan@cfsa.net.cn (X.P.); lining@cfsa.net.cn (N.L.); gych@cfsa.net.cn (Y.G.); liuxiumei@cfsa.net.cn (X.L.); yanlin@cfsa.net.cn (L.Y.); liying@cfsa.net.cn (Y.L.); yangshuran@cfsa.net.cn (S.Y.); hujing@cfsa.net.cn (J.H.); zhujianghui@cfsa.net.cn (J.Z.)

**Keywords:** microbiology, surveillance, food safety, China

## Abstract

Microbiological food safety surveillance is a system that collects data regarding food contamination by foodborne pathogens, parasites, viruses, and other harmful microbiological factors. It helps to understand the spectrum of food safety, timely detect food safety hazards, and provide relevant data for food safety supervision, risk assessment, and standards-setting. The study discusses the microbiological surveillance of food safety in China, and introduces the policies and history of the national microbiological surveillance system. In addition, the function and duties of different organizations and institutions are provided in this work, as well as the generation and content of the surveillance plan, quality control, database, and achievement of the microbiological surveillance of food safety in China.

## 1. Introduction

Food safety is one of the most important concerns of humans, and closely related to health, economic development, and social stability [1]. In recent years, multiple disease outbreaks attributed to microbiological hazards have occurred around the world. To date, developed countries have shifted the focus of food safety from investigating the cause of foodborne disease outbreaks post-occurrence to proactive detection and prevention of food contamination [2]. To reach this goal, some countries, such as Ireland [3] and the United States [4], have launched food microbiological surveillance programs. It is estimated that 56.1% of food poisoning outbreaks were caused by microorganisms in China in 2012 [5], therefore, it is very important to be fully acquainted with food microbiological contamination and control the risk factors to ensure food safety. This paper introduces microbiological surveillance of food safety in China for the first time.

## 2. Policy Support

In 2000, the Department of Health began the surveillance of foodborne pathogens according to the planning of the Global Environment Monitoring System-Food Contamination Monitoring and Assessment Program. As the supporting organization for national food safety, the Institute of Nutrition and Food Safety of China Center for Disease Control and Prevention commissioned some provincial Centers for Disease Control and Prevention to launch surveillance and establish the National Surveillance Network of foodborne pathogens based on a project from the Ministry of Science and Technology [6]. In August 2003, the Ministry of Health issued a document titled “Food Safety Action Plan”, which stressed the need to establish a surveillance network of foodborne pathogens and also incorporate the work into a special management by providing financial support every year [7].

The Food Safety Law of the People’s Republic of China was issued in 2009 and legally clarified the role and duties of the national food safety surveillance system for contamination and hazard factors in food [8]. One regulation was promulgated for the implementation of the Food Safety Law [9]. A revised version of the Food Safety Law of the People’s Republic of China was issued in 2015, which emphasizes the principle of risk prevention and improvement of the food safety surveillance system. The NHFPC conducts food safety surveillance jointly with the China Food and Drug Regulatory Department (CFDA). NHFPC, CFDA, and the General Administration of Quality Supervision, Inspection and Quarantine (AQSIQ) jointly develop and enforce the national food safety risk surveillance plan. Based on the national surveillance plan, the Provincial Health Department, jointly with the Food and Drug Regulatory Department and the Quality Supervision Department at the same level, shall formulate and adjust the food safety surveillance plan that takes into account the regional particularities. A technical institute shall carry out the food safety surveillance work pursuant to the surveillance plan to guarantee truthfulness and accuracy of the surveillance data. In the event that surveillance results reveal possible food safety risks, the county and above level health departments shall notify the information to the Food and Drug Regulatory Department, the government of the same level, and the higher level health department. The Food and Drug Regulatory Department shall then conduct further investigation [10].

## 3. History of National Microbiological Surveillance of Food Safety in China

Development of the microbiological surveillance system of food safety in China has passed through two stages. The first stage encompassed the surveillance of foodborne pathogens from 2000 to 2009. It aimed to evaluate food safety risk, develop policy and standards, and guide food hygiene supervision, production, and consumption. The number of participating provinces gradually expanded from 32.3% (10/31) in 2000 to 71.0% (22/31) in 2009, and prefecture-level cities covered from 10.5% (35/334) in 2000 to 51.2% (171/334) in 2009. In 2000 the surveillance only tracked contamination of *Listeria monocytogenes*, *Salmonella* spp., and *E coli* O157:H7 in over 1900 samples, including raw meat, cured meat, yoghourt, raw milk, ice cream, and aquatic products of animal origin for eating raw [6]. With time and hard work, the surveillance network achieved rapid development from 2000 to 2009. In 2009, the surveillance extended to two hygiene indicator bacteria and nine foodborne pathogens with over 19,000 samples, which included daily consumed foods by Chinese residents such as meat and meat products, eggs and egg products, milk and dairy products, animal foods, aquatic products, and vegetables.

The second stage was the establishment of the National Microbiological Surveillance Network in 2010. For the implementation of food safety surveillance required by the food safety law of the People’s Republic of China issued in 2009 [8], the National Microbiological Surveillance of Food Safety was launched in 2010 in all provinces, autonomous regions, and municipalities. Surveillance contents extended to food hygiene indicator bacteria, foodborne pathogens, viruses, and parasites in many food categories. In addition, sampling points were no longer limited to retail and catering places, and were broadened to farming, processing, and sales locations. A total of 99.1% (331/334) municipal-level institutions were involved in sample detection, and surveillance areas have covered about 86.8% (2484/2862) country-level government areas in 2014.

## 4. Functions and Duties of Organizations and Institutions in the National Microbiological Surveillance

**Figure 1:**
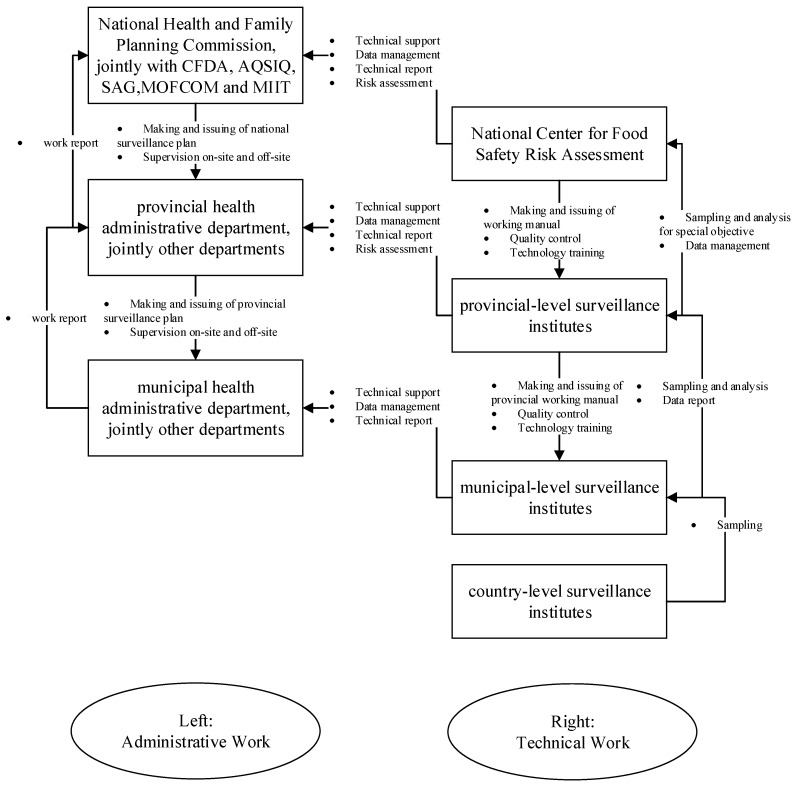
Flow chart of Microbiological food safety surveillance in China.

For efficiency, the national food safety surveillance plan was jointly issued by NHFPC, the Ministry of Industry and Information Technology (MIIT), the Ministry of Commerce (MOFCOM), the General Administration of Quality Supervision, Inspection and Quarantine (AQSIQ), the Food and Drug Administration (CFDA), and the State Administration of Grain (SAG), according to the Food Safety Law of the People's Republic of China and its implementation regulations (Figure 1).

The national microbiological surveillance program aimed to obtain the levels and trends of main microbiological contaminants of major foods in China, determine the potential sources and distributions of the hazards, provide a scientific basis for food safety risk assessment, standards setting, and tracking and evaluation; these actions help in taking appropriate risk control measures timely.

The China National Center for Food Safety Risk Assessment (CFSA) is responsible for providing technology guidance to the national food safety surveillance, personnel training and quality control; in addition, CFSA gathers, analyzes and manages the surveillance data, and identifies food safety problems and puts forward timely proposals. When the national surveillance is issued yearly, the CFSA gathers experts from epidemiology, microbiology, statistics, and quality control fields to draft and revise the manual guiding future surveillance work.

The provincial Health and Family Planning Administrative Departments must establish regional surveillance programs according to the national surveillance plan’s content and requirements. It should clearly appoint the technological institutions for implementation, management, trainings, analysis of the regional surveillance data, and notification and consultation of the surveillance results.

Food safety surveillance in China is very different from the European Union. In China, the national government pays most of the costs of annual food safety surveillance, and every province must complete all of the work according to the national surveillance plan, and, additionally, provide a real-time report of all detailed surveillance results to the central government [11,12]. An annual national surveillance plan is developed for the forthcoming year and issued in October before the implementation year. However, there is no a long term planning with sampling number, food category and contamination substances such as AVV Monitoring 2011–2015 in Germany [13], however, this concept is under consideration now according to the five-years surveillance result and risk assessment.

## 5. Surveillance Plan Generation and Content

The Health and Family Planning Commission issues an annual notice of advice and proposal to the National Food Safety Surveillance Plan from the Administration of Quality Supervision, Inspection and Quarantine, State Administration for Industry and Commerce, State Food and Drug Administration, and relevant research institutes and industry associations. CFSA is responsible for the summary and analysis of all concerns and suggestions, and invites experts from different departments involved in food safety supervision, industrial management, food inspection, and laboratory quality control for panel discussion. The resulting draft is proposed to related departments, and, after consultation, a final food safety surveillance plan is formed with the approval of all relevant departments. The surveillance items and content of the plan must be guided by the following principles:

(1) Foods with greatest health hazards and highest risks must be included;
(2) Food easily causing health effects on special groups such as infants and young children must be emphasized;
(3) Foods with wide and significant consumption should be taken into consideration;
(4) Foods and items ever involved in domestic food safety accidents in the past or with high attention by consumers must be included;
(5) Foods involved in health hazards abroad, with evidence of possible existence domestically must be included.

There are three types of microbiological surveillance: routine, special, and emergency surveillances. Routine surveillance includes a wide range of representative organisms to obtain the overall microbial contamination and its trend in foods, e.g., *Salmonella* in raw meat and *Vibrio parahaemolyticus* in fish. Special surveillance explores the contamination source and critical control points, which includes processing surveillance and surveillance of food chains, e.g., *Cronobacter* spp. in infant powdered formula processing surveillance and *Vibrio parahaemolyticus* in aquaculture, sales, and catering of freshwater fish. These two kinds of surveillance are closely related. For example, routine surveillance showed that the contamination level of *Vibrio parahaemolyticus* in freshwater aquatic products of animal origin is similar to seawater aquatic products of animal origin, and special surveillance was initiated to trace the source of *Vibrio parahaemolyticus* in freshwater fish. The surveillance of the food chain enables detection of hazards so that systematic control and intervention strategies can be adopted. Emergency surveillance is a quick-start process used only in the event of food safety emergency incidents, when unplanned risk assessment is needed.

## 6. Quality Control

To ensure scientific and accurate sampling and data analysis, and to improve the ability of surveillance institutions, CFSA organizes technological trainings in sampling, examination methods, and data reporting every year. Provincial-level institutes should train the municipal-level counterparts with the same contents, and amplify the training contents according to the actual situation, e.g., internship in the laboratory. There is detailed requirement for the design of a concrete implementation plan for sampling, quality control, analysis methods, information reporting, and data management in the annual manual of surveillance.

Surveillance work in every institute should incorporate a quality management system. CFSA organizes and develops methods for quality control, for example, to distribute quality control analysis samples for examination, evaluate data quality and work performance yearly in each province, and communicate at the annual work conference and training meeting the outstanding experiences. Provincial-level institutes should be responsible for the improvement of municipal-level institutes with wrong examination results and other mistakes.

All these data are submitted to CFSA by the Network System of National Food Microorganism surveillance [3]. In order to ensure data quality and timeliness, the data should be audited and submitted in sequence from the municipal-level institutes, then to provincial-level institutes, and finally to CFSA within five days of obtaining the analytical results. They will be valid data in the national database after a three-level audit system. On the other hand, provincial-level institutes should review the positive isolation of foodborne pathogens to ensure the accuracy of surveillance results. The accuracy and timeliness of data reporting and result analysis are important indicators in evaluating the work performance of the different provinces. Through continuous technical training and exchange of experience, the surveillance capability is evolving and improving rapidly.

## 7. Microbiological Surveillance Database

There are over two million pieces of data from about 500,000 samples, including various kinds of major foods and local foods related to foodborne diseases. Surveillance items include mainly foodborne pathogens such as *Salmonella, Cronobacter* spp*., Bacillus cereus, Staphylococcus aureus, Listeria monocytogenes, Vibrio parahaemolyticus,* *Campylobacter jejuni* and *Clostridium botulinum*, as well as hygiene indicator bacteria such as *E. coli* and *other Enterobacteriaceae.* Parasites are relatively less monitored, and mainly include nematodes, *Angiostrongylus cantonensis*, and *Trichina* spp. The only virus monitored is Norovirus. The information database of food microbial contamination in China has been established, which gradually clarifies food contamination of main foodborne pathogens in different regions, seasons, packaging types, food categories, brands, and sampling sites in China. The contamination data regarding main foodborne pathogens and indicator bacteria are well representative, but it is still necessary to strengthen the surveillance of foodborne viruses, parasites and several pathogens due to the fact that only part of the laboratories can perform an analysis. For example, *Campylobacter jejuni* is one of the world’s most important food poisoning pathogens [14], however, there has been relatively little data collected about the incidence of food contamination and foodborne disease because only some laboratories can do the analysis of this pathogen-resulting in very different occurrence rates from 0.5% to 20% in raw chicken meat from various Chinese provinces.

## 8. Achievement of the Microbiological Surveillance

The nationwide network and system for food microbiological surveillance is the responsibility of CFSA, who acts as the technical leader in this work, the provincial and prefecture level institutions as technical support and backbone, respectively.

Through continuous surveillance and accumulating scientific data, the basic contamination levels and trends in major foods, such as meat and meat products, milk and dairy products, eggs and egg products, animal products, cooked rice flour products, infant and young children foods, and many other kinds of Chinese meals, are now clarified. The current monitoring results show that the overall situation of relevant foods is good, although some food safety problems remain. The microbiological contamination level in bulk products is higher than that of prepackaged products. However, foodborne pathogens were still isolated from prepackaged products, especially those produced by contract manufacturers and small businesses. Seasonal differences also occur, with higher detection levels in the second and third quarters for microbial contamination of bulk products, but not prepackaged products. Microbiological contamination is higher in street stalls, especially unlicensed mobile stalls when compared with other sampling sites. However, there are exceptions. For example, bulk ice cream at a large hotel has higher microbiological risk than that served in the fast-food cafe, perhaps due to the length of shelf life in the big hotel. Cross contamination during food processing is common; for example, fruit salad is higher risk than whole fruit, and *Salmonella* spp. and *Listeria monocytogenes* often contaminate cooked meat. In addition, the contamination rate of *Salmonella* spp. in raw chicken is higher than that in live chicken, due to cross contamination in the slaughterhouse. As favorite and delicious foods of many Chinese, raw aquatic products pose a high risk for foodborne diseases because of *Vibrio parahaemolyticus* and *listeria monocytogenes*.

Food surveillance provides basic data and technical support for risk assessment and standards-setting, and promulgation of food safety laws. The surveillance results provide advices and tips for assessment projects, relevant analysis methods, and standards of food safety. For example, the data regarding *Listeria monocytogenes* in ready-to-eat food have been used in the food safety risk assessment project “quantitative risk assessment of *Listeria monocytogenes* in ready-to-eat food, where suggestions were provided to the classification and revision of “National Food Safety Criteria For Foodborne Pathogen” (GB 29921-2013) [15]. According to surveillance results of *Bacillus cereus* in powdered infant formula from 2011 to 2013 [16] and criteria from European Union [17], Australia, and New Zealand [18], the relevant assessment project was promoted to begin a risk assessment in China to evaluate the potential hazard of *Bacillus cereus* contamination in powdered infant formula and the necessity of setting-up relevant microbiological criteria.

To give full play to the role of food safety surveillance, CFSA timely analyzes the reported results, immediately organizes examination when necessary, and identifies possible potential hazards based on timely and accurate principles. For example, the control of production process of powdered infant formula was initiated from 2013 to 2014 to find the contamination source due to persistent contamination of retail products from 2011 to 2012. The contamination of foodborne pathogens decreased greatly in the infant powdered formula by this two-year action, and the incidence of *Cronobacter* spp. occurrence in this retail product decreased rapidly from 1.1% in 2012 to 0.35% in 2013. Additionally, the surveillance system has changed the focus of food safety priorities. For example, the notification of contamination by *Cronobacter* spp. and *Bacillus cereus* in goat powdered infant formula provided clues for the supervision and administration of this kind of food. A 0.94% and 2.51% occurrence of *Salmonella* spp. and *Listeria monocytogenes* in bulk packed cooked meat products in the Chinese retail market is higher than these in the pre-packed cooked meat with an occurrence of 0.03 % and 0.28 %. However, as one of the most popular foods for Chinese and one of the most common sources of food poisoning in China, there is no microbiological food safety criteria for bulk packed cooked meat in China, which has caused particular attention of the government and highlights that the next step of risk assessment and criteria development is necessary.

## 9. The Future of the Microbiological Surveillance

The national microbiological surveillance data only reflect the overall Chinese food safety situation, but not special aspects of every province, autonomous region, and municipality. The sample numbers are relatively low to complete a comprehensive and systematic risk assessment at the provincial level. Therefore, it is recommended to establish a local investment and safeguard mechanism to develop local microbiological surveillance, especially for local characteristic foods.

It is very important to apply information technology in the development of surveillance. Currently, the value and significance of spatial analysis technology of a Geographic Information System in the application of risk analysis and early warning is being explored. An exchange platform will be built to share the national food safety surveillance data between different organizations and institutions. These advances in data security, data analysis, and early warning will improve the timeliness of data utilization and response. In addition, the use of network information will become an important field of food safety risk assessment, with the continuous advance of the "big data" era on the internet.

## 10. Conclusions

It’s been proved that surveillance system is a powerful tool for increasing food safety. The Chinese government has worked hard to build a comprehensive surveillance system, but there is still much work that needs to be done despite the rapid development over the past six years.
